# Os-Artificial Impugned

**Published:** 1869-11

**Authors:** 


### OS-ARTIFICIAL IMPUGNED.

"To me, there is nothing reasonable in the idea that oxychloride of zinc can induce the formation of natural bone over exposed pulps."—Contributor "Miscellanies," October REGISTER.

There are many things on this sublunary sphere beyond the erudite comprehension of finite mortals; and the paragraph quoted above is only another illustration of this fact. "To me," it is always a healthy sign to observe a man's reasoning powers in the natural process of exercise, if, peradventure, he fails to reconcile his ideas. To have incompatible elements clog or retard the genial and even-tenor of one's ideas, tends to excite sympathy—commiseration.

Desiring to aid this "Miscellaneous" contributor to a more favorable consideration of what he deems irreconcilable, it will be the aim of this paper to clear him up a little.

Among active members of the Dental profession in the discussions of our societies, and in the pages of our periodicals, os-artificial has received no small share of attention, and it was presumed that every practitioner was tolerably well informed with regard to its properties and merits. But amid all this blaze of intelligence there seems to be one, perhaps there are more, whose reason is so nicely poised that he can't be made to kick the beam, forego all scruples, and believe in the paradoxical os-artificial as a pulp protective.

A thing is reasonable or unreasonable just in proportion as our information is co-extensive. If our acquaintance be meager or limited; if we have not pushed our inquiries to a point of thoroughness, and thereby enable us to see clearly, then our account of things unreasonable will always be in the ascendancy. It is more than probable the contributor of "Miscellanies" has never patiently and carefully noted operations made of os-artificial over exposed pulps, beyond those made by himself. It is neither wise nor prudent to condemn an agent which, in the hands of dextrous and skillful operators, proves excellent in results. Such a course is too sweeping and immodest.

That there are multiplied cases where oxy-chloride of zinc has been placed over exposed pulps, and under this protective covering, in the course of time, an osseous deposit was thrown out at the point exposed, there is an abundance of testimony to the support of this fact.

The objections to os-artificial lie chiefly in the supposed escharotic property of the zinc chloride which is assumed to destroy the pulp, or lulling it into a repose whence there is no waking. If such were the fact, and no real difficulties of a periosteal inflammation, and the color of the teeth well preserved afterwards, what harm has it done? Will root filling answer any better purpose? But it should be remembered zinc chloride in the form it is used by us, is diluted, and scarcely possesses any escharotic property. When combined with the oxyd and applied to the exposed pulp without any intermediate protection, there is a slight tinge of pain following which lasts for a short time only. And this difficulty may be avoided by applying gently over the bare pulp after excavation is made, a coating of styptic colloid, which has a soothing and conservative influence. The positive failures in the use of os-artificial is more in the Dentist than in the agent. Nor is it reserved for a large number to achieve success while manipulations are so carelessly or heedlessly made. It requires a nicety of hand, and a delicacy of touch to prevent irretrievable injuries, that we may expect to hear this agent condemned and in disfavor. Its popularity is steadily gaining. Its uses are daily multiplying. Who would be without it? Who does not feel proud daily, to see his failures rise into successes by its use. Let us return our gratitude to those who are our benefactors in bringing this material into Dental practice, and showing us how to use it.

